# Antibacterial Activity and Membrane-Disruptive Mechanism of 3-*p*-*trans*-Coumaroyl-2-hydroxyquinic Acid, a Novel Phenolic Compound from Pine Needles of *Cedrus deodara*, against *Staphylococcus aureus*

**DOI:** 10.3390/molecules21081084

**Published:** 2016-08-18

**Authors:** Yanping Wu, Jinrong Bai, Kai Zhong, Yina Huang, Huayi Qi, Yan Jiang, Hong Gao

**Affiliations:** 1 Department of Food Science and Technology, College of Light Industry, Textile and Food Engineering, Sichuan University, Chengdu 610065, China; wyp9202@163.com (Y.W.); baijinrong01@hotmail.com (J.B.); eric211@163.com (K.Z.); 2 Department of Public Health, West China Medical School, Sichuan University, Chengdu 610041, China; 3 Chengdu Institute of Biology, Chinese Academy of Sciences, Chengdu 610041, China; qihy@cib.ac.cn (H.Q.); jiangyan@cib.ac.cn (Y.J.)

**Keywords:** phenolic compound, *Staphylococcus aureus*, antibacterial activity, antibacterial mechanism, membrane damage

## Abstract

Recently, we reported that a novel phenolic compound isolated from *Cedrus deodara*, 3-*p*-*trans*-coumaroyl-2-hydroxyquinic acid (CHQA), exhibits a potent antioxidant activity. The present study aimed to evaluate the antibacterial activity of CHQA against eleven food-borne pathogens and to elucidate its mechanism of action against *Staphylococcus aureus*. The results from minimum inhibitory concentration (MIC) determinations showed that CHQA exhibited moderate inhibitory effects on all of the tested pathogens with MIC values ranging from 2.5–10 mg/mL. Membrane potential measurements and flow cytometric analysis demonstrated that CHQA damaged the cytoplasmic membrane of *S. aureus*, causing a significant membrane hyperpolarization with a loss of membrane integrity. Moreover, CHQA induced an increase in membrane fluidity and conformational changes in membrane protein of *S. aureus*, suggesting that CHQA probably acts on the cell membrane by interactions with membrane lipid and protein. Transmission electron microscopic observations further confirmed that CHQA disrupted the cell membrane of *S. aureus* and caused severe morphological changes, which even led to leakage of intracellular constituents. These findings indicated that CHQA could have the potential to serve as a natural antibacterial agent to control and prevent the growth of pathogens in food and in food-processing environments.

## 1. Introduction

Food-borne diseases are one of the most important issues that continue to be a major concern for the food industry and consumers worldwide, even in well-developed countries [1,2]. Predominant pathogens causing food-borne diseases include *Salmonella* sp., *Staphylococcus aureus*, *Clostridium perfringens*, *Bacillus cereus*, *Escherichia coli*, and *Vibrio parahaemolyticus* [2,3]. Among them, *S. aureus* is a significant pathogen that can cause a series of food-borne diseases, ranging from mild skin infections to severe and potentially fatal diseases such as endocarditis, osteomyelitis, and pneumonia [4]. With the ability to survive and grow in a wide range of environmental conditions, *S. aureus* has been found in various types of foods, such as meat, egg, milk, salads, and bakery products, which imposes high risk to human health [2,5]. Therefore, it is important to control and prevent the growth of this pathogen in food and in food-processing environments.

Although the application of physical techniques and chemical preservatives can effectively prevent the growth of pathogenic and spoilage microorganisms in food, their negative effects on the sensitive nutrients, organoleptic properties, and human health are receiving growing attention [6,7]. Meanwhile, with the ever-increasing demand of consumers for minimally processed, nutritional, safe, and natural foods, considerable effort has been made to search for efficient natural antimicrobials as safer preservative alternatives. In recent years, phenolic compounds derived from plants have been extensively screened due to their antimicrobial activities against a broad range of food spoilage and food poisoning microorganisms [8].

**Figure 1:**
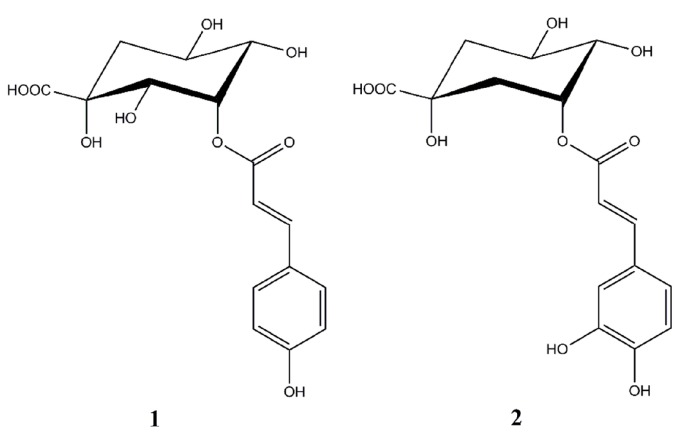
Chemical structures of 3-*p*-*trans*-coumaroyl-2-hydroxyquinic acid (CHQA, **1**) and chlorogenic acid (**2**).

3-*p*-*trans*-Coumaroyl-2-hydroxyquinic acid (CHQA, Figure 1) is a novel phenolic compound, firstly isolated from pine needles of *Cedrus deodara*, which showed a potent antioxidant activity in both radical scavenging capacity and cell-based oxidative haemolysis inhibition assay in our previous study [9]. CHQA is a unique natural compound bearing a 2-hydroxyquinic acid moiety together with an ordinary *p*-coumaric acid moiety. Lou et al. have demonstrated that *p*-coumaric acid killed bacteria by disrupting bacterial cell membranes and binding to bacterial genomic DNA to inhibit cellular functions [10]. It is worth noting that 5-*p*-*cis*-coumaroylquinic acid was reported to exhibit moderate antibacterial activities against five common food-borne pathogens [11]. Moreover, the chemical structure of CHQA is similar to that of chlorogenic acid (**2**, Figure 1), which is a common antimicrobial agent [12,13]. However, there is no available report related to the antibacterial activity of CHQA against food-borne pathogens so far. Bearing this in mind, the aims of the present study were to evaluate the antibacterial activity of CHQA against several common food-borne pathogens by measuring the minimum inhibitory concentration (MIC) values, and to further investigate the possible mechanism of action against *S. aureus* by determining the effects of CHQA on membrane potential, membrane integrity, membrane fluidity, membrane protein, and cell morphology.

## 2. Results

### 2.1. Antibacterial Activities of CHQA

The antibacterial activities of CHQA and chlorogenic acid against various bacterial strains were compared and described with MIC values in Table 1. The MIC values of CHQA and chlorogenic acid for the tested bacterial strains were both in the range of 2.5–10 mg/mL, and the majority of the strains showed medium sensitivity with MIC of 5 mg/mL. Among these bacteria, both compounds showed the most prominent effect on *B. cereus*, with the minimum MIC of 2.5 mg/mL, while the lowest inhibitory activity was noticed for *E. coli*, with the maximum MIC of 10 mg/mL. The results showed that CHQA exhibits moderate inhibitory effects on all of the tested food-borne pathogens, including both Gram-negative and Gram-positive bacteria, which was comparable to that of chlorogenic acid. Considering the serious harmfulness and widespread occurrence of *S. aureus*, it was chosen to be the representative for the further mechanism investigation in this study.

### 2.2. Effect of CHQA on Membrane Potential

**Figure 2:**
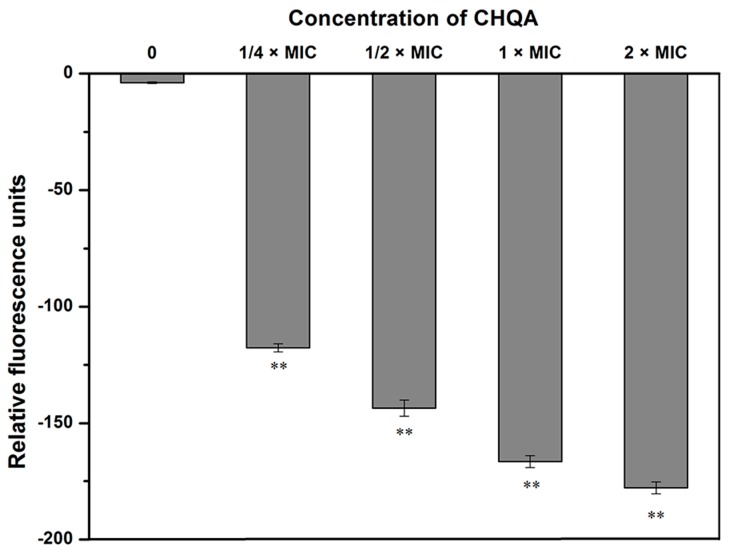
Effect of CHQA on the membrane potential of *S. aureus* ATCC 6538. Bars represent the standard deviation (*n* = 3). ** *p* < 0.01.

The change in the membrane potential of *S. aureus* ATCC 6538 cells after treatment with CHQA was evaluated using DiBAC_4_(3). As shown in Figure 2, the fluorescent intensity of untreated *S. aureus* was detected to be −3.95 ± 0.26, while the addition of CHQA at 1/4 × MIC caused a significant decrease (*p* < 0.01) in fluorescence intensity from −3.95 ± 0.36 to −117.66 ± 1.77. Additionally, a further decrease in membrane potential was observed when the concentration of CHQA increased from 1/4 × MIC to 2 × MIC, revealing that CHQA caused a significant hyperpolarization of the *S. aureus* cytoplasmic membrane in a dose-dependent manner.

### 2.3. Effect of CHQA on Membrane Integrity

**Figure 3:**
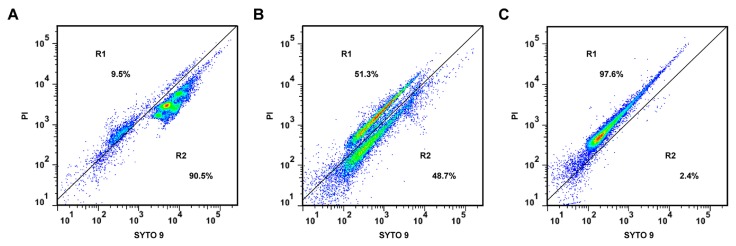
Flow cytometric analysis of SYTO 9-PI (propidium iodide)-stained *S. aureus* ATCC 6538. (**A**) untreated; (**B**) treated with CHQA at 2 × MIC for 3 h; (**C**) treated with 70% isopropyl alcohol for 3 h. Regions R1 and R2 represent the membrane damaged or dead cells and live cells, respectively.

The loss of membrane integrity of *S. aureus* ATCC 6538 treated with CHQA was assessed by measuring the fluorescent signals from SYTO 9 and propidium iodide (PI) with a flow cytometer. SYTO 9 emits green fluorescence and is able to stain both live and dead cells, whereas red fluorescent PI can only penetrate bacteria with damaged membranes, causing a reduction in the fluorescence intensity of SYTO 9 when both stains are present [14]. As a result, bacterial cell populations on the dot plots were clustered in two different blocked regions: R1 and R2 (Figure 3). R1 corresponds to strong red fluorescence, which indicates the dead or membrane-damaged cells. On the other hand, R2 reflects strong green fluorescence, representing live cells. After 3 h exposure to 2 × MIC of CHQA (Figure 3B), the percentage of cells with intact membrane markedly decreased from 90.5% to 48.7% compared with the negative control (Figure 3A), whereas that only accounted for 2.4% following treatment with 70% isopropyl alcohol (Figure 3C). The results demonstrated that CHQA exposure induced damages in the cell membrane of *S. aureus* by a loss of membrane integrity.

### 2.4. Effect of CHQA on Membrane Fluidity

**Figure 4:**
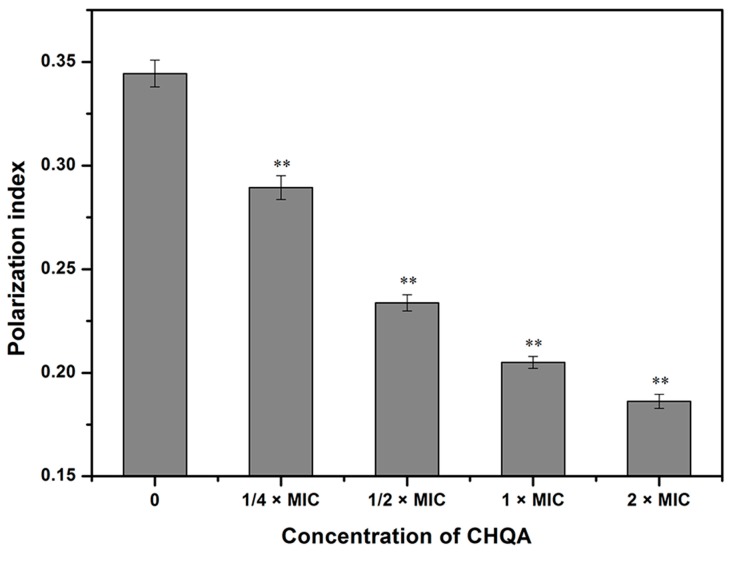
Effect of CHQA on the membrane fluidity of *S. aureus* ATCC 6538. Bars represent the standard deviation (*n* = 3). ** *p* < 0.01.

Further antibacterial mode of action of CHQA against *S. aureus* ATCC 6538 was confirmed using an assay of cell membrane fluidity. As shown in Figure 4, the fluorescence polarization values of 1,6-diphenyl-1,3,5-hexatriene (DPH) in *S. aureus* cells treated with CHQA at 1/4 × MIC, 1/2 × MIC, 1 × MIC, and 2 × MIC were 0.289 ± 0.006, 0.234 ± 0.004, 0.205 ± 0.003, and 0.186 ± 0.003, respectively, whereas the control cells had a fluorescence polarization value with 0.344 ± 0.006. As a consequence, the fluorescence polarization of DPH decreased significantly (*p* < 0.01) with the increasing concentration of CHQA from 1/4 × MIC to 2 × MIC, implying that CHQA caused increasing membrane fluidity.

### 2.5. Effect of CHQA on Membrane Protein

**Figure 5:**
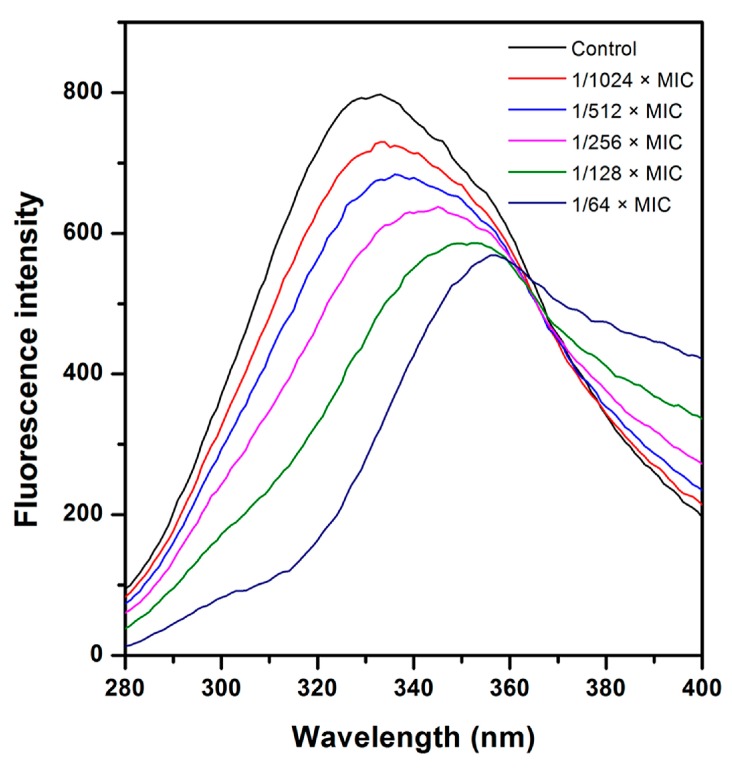
Effect of CHQA on fluorescence intensity of the *S. aureus* ATCC 6538 membrane protein at λ_ex_ 258 nm.

The effects of CHQA on the fluorescence spectra of the membrane protein of *S. aureus* ATCC 6538 were shown in Figure 5. There was a maximum fluorescence peak at 333 nm with the fixed excitation wavelength at 258 nm, which mainly belongs to the Phe residues of membrane protein. However, after incubation with CHQA, the fluorescence intensity was quenched gradually with the increasing concentration of CHQA. In addition, the fluorescence peak showed a significant red shift accompanied by an increase in the fluorescence emission in the larger wavelength region, which may indicate that the binding of CHQA to the membrane protein changed the microenvironment of Phe residues along with energy transfer between CHQA and membrane protein [15,16]. Consequently, the spectral change herein reflected the corresponding change in conformation and structure of *S. aureus* membrane protein after interacting with CHQA.

### 2.6. Effect of CHQA on Cell Morphology

**Figure 6:**
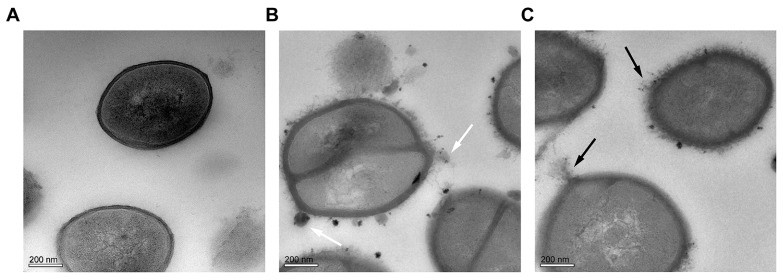
Transmission electron microscopy images of *S. aureus* ATCC 6538. (**A**) untreated bacteria; (**B**) and (**C**) bacteria treated with CHQA at 2 × MIC for 6 h.

TEM experiments were carried out to directly observe membrane damage and ultrastructure change induced in *S. aureus* ATCC 6538 cells following exposure to CHQA (Figure 6). The untreated cells displayed a normal morphology and had a smooth and compact cell membrane with homogeneous electron density in the cytoplasm. In contrast, the bacterial cells incubated with CHQA at 2 × MIC revealed detrimental effects on the morphology of the cell wall and the cytoplasmic membrane, showing uneven envelope, lysis of membrane integrity and leakage of intracellular contents (black arrows). In addition, small amounts of condensed substances or granular agglutination around the cell membrane were observed in the *S. aureus* cells treated with CHQA (white arrows).

## 3. Discussion

The survival and growth of harmful microorganisms in food have been considered the primary causes of food quality deterioration and food-borne diseases. Food preservation plays an important role in the food industry, which is widely applied to ensure food safety and extend the shelf life of food products. Recently, antimicrobial and various other bioactivities of plant second metabolites, coupled with the increasing negative awareness of consumers on synthetic preservatives have promoted the food industry to search for natural preservatives. Phenolic compounds have been reported to possess strong antioxidant and antimicrobial activities, which are beneficial for the prevention of rancidity and spoilage in high fat and lipid-based foods and restrict the growth of undesirable microorganisms [17].

In the present study, the results from the MIC determination revealed that CHQA had moderate and consistent inhibitory effects against all of the tested food-borne pathogens, with different susceptibility rates in different bacteria (Table 1). It is worth noting that the Gram-negative *E. coli* exerted the most resistance to CHQA, while the Gram-positive *B. cereus* were most sensitive in the antibacterial experiments. This observation was probably related to the significant differences in cell membrane structure and composition between Gram-positive and Gram-negative bacteria. The resistance of *E. coli* toward CHQA could be attributed to the thick layer of lipopolysaccharide outer membrane, whereas the cell membrane of *B. cereus* is easier to weaken because of the single peptidoglycan layer structure [18]. Moreover, for all of the tested pathogens, the MIC values of CHQA were the same as that of chlorogenic acid. However, some studies have demonstrated that the number and position of hydroxyl groups could play a significant role in the antimicrobial activity of phenolic compounds [19,20]. Specifically, it was reported that caffeic acid showed higher antimicrobial activity compared to *p*-coumaric acid due to the addition of one more hydroxyl group at the phenolic ring [21]. So, it was implied that the additional hydroxyl group in the 2-hydroxyquinic acid moiety of CHQA might contribute to the antibacterial activity. However, further studies are required to reveal the antibacterial activity of CHQA in relation to the position and number of coumaroyl group attached to a 2-hydroxyquinic acid or a quinic acid core.

Diverse phenolic compounds are generally believed to principally perform their antibacterial mechanism against the cytoplasmic membrane of bacterial cells, which is mainly attributed to the presence of hydroxyl groups [1]. The accumulation of hydrophobic phenolic groups in the lipid bilayer may disrupt lipid–protein interaction and increase membrane permeability, further causing alterations in membrane structure and accelerating the extensive leakage of intracellular constituents, finally destroying membrane integrity to facilitate the entry of more antibacterial agents [22]. Nevertheless, it was not clear whether the antibacterial mechanism of CHQA—which possesses its own special chemical structure of a 2-hydroxyquinic acid moiety and ester group—was same or different. In the present study, the mode of action of CHQA against *S. aureus* was elucidated by investigating changes in cell surface characteristic parameters (including membrane potential and membrane integrity), interactions of CHQA with membrane components such as lipid and protein, and alterations in cell morphology and ultrastructure.

Membrane potential alteration is an early indication of injury in bacteria, and can be evaluated by measuring the fluorescent intensity of DiBAC_4_(3). DiBAC_4_(3) is an anionic, voltage sensitive fluorescent probe that crosses the cytoplasmic membrane according to the transmembrane potential, with low intracellular fluorescence indicating the hyperpolarization of a cell [23]. The results showed that CHQA caused membrane hyperpolarization of *S. aureus* cells, as evidenced by a decrease in fluorescence (Figure 2). Consistent with our findings, a recent study reported that chlorogenic acid could damage the cell membrane of *S. aureus*, causing membrane hyperpolarization [12]. Previous studies of this phenomenon have suggested that hyperpolarization occurs primarily due to a pH change or increased movement of ions, specifically K^+^, which diffuse outside to balance membrane potential [24]. Moreover, the maintenance of ion homeostasis is integral to cell growth, which is crucial for numerous energy-relevant cellular processes, including solute transport, regulation of metabolism, management of turgor pressure, and control of motility [25]. Thus, the perturbation of membrane potential induced by CHQA may affect the overall cell metabolism, eventually leading to cell death.

Numerous studies have reported that phytochemicals possess a membrane-active mechanism that causes severe membrane damage through the disruption of the membrane integrity. Therefore, membrane integrity was chosen as another parameter to explore the antibacterial mechanism of CHQA. The results showed that exposure of *S. aureus* to CHQA increased the proportion of cells in the PI fluorescent region (Figure 3), indicating an increase in the number of membrane-damaged bacterial cells. It is noteworthy that the majority of cell populations in the R2 region of the CHQA-treated sample displayed weaker green fluorescence than that of the untreated sample. These cell populations may represent potentially injured bacteria or intermediate state bacteria, suggesting that CHQA may influence the physiological status of the *S. aureus* cells which were detected to even remain impermeable to PI stain. Although combination of SYTO 9 and PI does not always differentiate distinct live and dead populations, this technique is considered to provide a good estimate of the membrane integrity of various bacteria [14]. Membrane integrity is a crucial factor to the barrier function of the cell membrane, which plays an important role in maintaining optimal internal conditions for metabolism and energy transduction [26]. Thus, even relatively slight damage to the structural integrity of the cell membrane could detrimentally affect cell metabolism, resulting in cell growth inhibition and even death [25]. The results herein revealed that CHQA caused a significant disruption on the membrane integrity of *S. aureus*, which was consistent with the results of the membrane potential assay, indicating that the cell membrane could be the primary target of CHQA in its antibacterial action. However, it is still unclear which biomolecules on the cell membrane are being targeted by CHQA—membrane lipid or membrane protein.

Interactions between chemicals and membrane lipids may give rise to dramatic effects on membrane fluidity, which mainly reflects the order, shape, packing, and curvature of membrane lipids [27]. Therefore, changes in membrane fluidity were determined as an indicator for the assessment of the effect of CHQA on membrane lipids. DPH is a hydrophobic fluorescent probe which can intercalate between the phospholipids of the cell membrane, and emits fluorescence depending on its environment [28]. The fluorescence polarization of DPH reflects the structural order of membrane lipids, and a decrease of the polarization index indicates an increase of membrane fluidity [28]. The results showed that CHQA significantly increased the membrane fluidity of *S. aureus* cells in a dose-dependent manner (Figure 4). This is the possible evidence that CHQA could incorporate into the cytoplasmic membrane lipid bilayers and even alter the structural function of the membrane. Similar to our findings, some essential oils were demonstrated to damage the cell membrane of bacteria and increase the fluidity of the lipid bilayer core of the membrane [29]. It was also reported that natural phenolic compounds might integrate into the monolayers which are composed of bacterial phospholipids, thus increasing the membrane fluidity [30]. Moreover, it is noteworthy that the modification of fluidity can increase the permeability of the cell membrane and lead to leakage of cellular contents, which might consequently affect numerous other cellular processes [29].

Integral and peripheral membrane proteins—which constitute an important component of bacterial cytoplasmic membrane—provide various cell functions, including nutrient transportation, enzymatic activity, and transfer of cellular information. Therefore, analyzing the interaction of CHQA with membrane proteins may give further insight into the mechanism of action against the bacterial cell membrane. As shown in Figure 5, the significant changes in the fluorescence spectra clearly demonstrated that CHQA changed the conformation and structure of the *S. aureus* cell membrane proteins and may make the Phe residues located inside the membrane exposed outside of the membrane [31]. Subsequently, these membrane protein conformational changes would inhibit multiple related functions and further influence the interplay between host and pathogen, potentially reaching a critical level to cause the loss of bacterial viability [32]. Interestingly, although shikimic acid was also found to be capable of quenching the fluorescence of Phe residues of *S. aureus* membrane protein in our previous study [33], the quenching spectra for CHQA were different from that for shikimic acid, with a concomitant increase in the fluorescence emission in the larger wavelength region. This implied that the two compounds might interact with the membrane protein of *S. aureus* by different binding properties, due to their different molecular structures. Nevertheless, further studies based on isolated bacterial membrane protein may be required to understand the detailed binding mode of CHQA. Combined with the results of the membrane fluidity assay, it was implied that membrane lipids and membrane proteins could be the target molecules on the cell surface for the action of CHQA against *S. aureus*.

The TEM observation of *S. aureus* cells treated with CHQA showed that severe morphological modifications appeared in cell wall and membrane, which even led to leakage of intracellular dense materials on the cell surface (Figure 6). These results were in accordance with the results obtained from the membrane potential assay (Figure 2) and the membrane integrity assay (Figure 3). The findings are in good agreement with the previous report about the disruptive effect of chlorogenic acid on the *S*. *aureus* cell membrane [12]. Many previous transmission electron microscopy analyses showed that the effects of antimicrobial natural products on the bacterial cell morphology were various, including the formation of pores on the cell membrane [3], leakage of cytoplasmic materials [33], appearance of ghost-like bacteria, as well as complete cell lysis [34]. In the case of CHQA, lysis of the cell envelope and leakage of cytoplasmic contents were observed in the treated *S. aureus* cells. Moreover, the tightly condensed substances or dense granules accumulated around the cell surface were assumed to be the deposition of the cytoplasmic proteins of inactivated bacteria [35]. In this regard, CHQA might target different parts of the bacterial cell, and its antibacterial activity could not be ascribed to one specific mechanism. Therefore, further research investigating the effect of CHQA on intracellular macromolecules or bacterial pathways could provide additional information to fully understand the mode of action of CHQA.

## 4. Materials and Methods

### 4.1. Materials and Chemicals

CHQA (HPLC purity ≥ 98%) was purified from pine needles of *C. deodara* according to the previous reported method [9] and identified by Chengdu Institute of Biology, Chinese Academy of Sciences, Chengdu, China. The chemical structure of CHQA was shown in Figure 1. CHQA has good water solubility, and stock solutions were prepared in distilled water for use in all experiments. Chlorogenic acid (HPLC purity ≥ 98%) was purchased from Sigma Aldrich Co. (St. Louis, MO, USA). Nutrient broth, tryptone soy broth, and Mueller Hinton broth were purchased from Beijing Aoboxing Biotech Co. Ltd. (Beijing, China). All other chemicals used were of analytical grade.

### 4.2. Bacterial Strains and Culture Conditions

The antibacterial activities of CHQA were evaluated against eleven different food spoilage and poisoning microorganisms. Three Gram-negative strains, including *Escherichia coli* ATCC 11229, *Salmonella enterica* ATCC 6539, and *Vibrio parahaemolyticus* ATCC 17802, and eight Gram-positive strains, including *Bacillus cereus* ATCC 14579, *Clostridium perfringens* ATCC 13124, and five *Staphylococcus aureus* strains (ATCC 6538, ATCC 25923, ATCC 29213, ATCC 27217, ATCC 29247, and ATCC 9144) were obtained from the China Medical Culture Collection Center (Beijing, China). *V. parahaemolyticus* were inoculated into tryptone soy broth, and the other bacteria were inoculated into nutrient broth. Subsequently, the bacterial strains were cultured overnight at 37 °C with shaking at 130 rpm to obtain cells in the logarithmic phase.

### 4.3. Determination of the Minimum Inhibitory Concentration (MIC)

The MIC values of CHQA and chlorogenic acid for eleven different bacterial strains were determined by broth microdilution method with minor modifications [36]. Briefly, bacterial cells were collected in the logarithmic phase and diluted in Mueller Hinton broth to about 1 × 10^6^ CFU/mL. Aliquots of 100 μL of serial twofold dilutions of test compound in Mueller Hinton broth were added to individual wells of a sterile 96-well microplate in triplicate. Then, 100 μL of the bacterial suspensions were added to each well to achieve an inoculum of approximate 5 × 10^5^ CFU/mL. The plate was incubated at 37 °C for 24 h. The MIC value was defined as the lowest concentration of compound preventing visible growth of bacteria.

### 4.4. Membrane Potential

The experiment was carried out according to the method of Sánchez, García, and Heredia [26] with some modifications. In brief, the overnight culture of *S. aureus* ATCC 6538 was diluted with fresh nutrient broth to obtain a cell density of 1 × 10^7^ CFU/mL. The bacterial suspensions were subjected to treatment with different concentrations of CHQA at 25 °C for 10 min. Then, the treated bacterial suspensions were further incubated with 0.5 μg/mL of the membrane potential-sensitive fluorescent probe bis-(1,3-dibutylbarbituric acid) trimethine oxonol (DiBAC_4_(3); Life Technologies, Eugene, OR, USA) in the dark for 5 min. After incubation, fluorescence intensity of DiBAC_4_(3) was measured using a fluorescence spectrophotometer (Cary Eclipse G9800A, Agilent technologies trading Co., Ltd., Shanghai, China) at excitation and emission wavelengths of 492 nm and 515 nm, respectively. Background fluorescence resulting from CHQA added to the medium was determined and corrected.

### 4.5. Flow Cytometric Analysis

The effect of CHQA on the membrane integrity of *S. aureus* ATCC 6538 was investigated using the LIVE/DEAD BacLight bacterial viability kit (Life Technologies), as previously reported [37]. Briefly, logarithmic phase *S. aureus* cells were harvested by centrifugation at 3000 rpm for 5 min, washed, and resuspended at 1 × 10^9^ CFU/mL in 0.85% sterile saline. The bacterial suspension was incubated with 2 × MIC of CHQA at 37 °C for 3 h. After treatment, cells were washed and subsequently adjusted to 1 × 10^6^ CFU/mL with 0.85% sterile saline. Then, 1 mL of the suspension was incubated with 60 μM propidium iodide (PI) and 10 μM SYTO 9 for 15 min in the dark at room temperature. The flow cytometric analysis was performed by running the suspensions on a BD FACSVerse flow cytometer (Becton Dickinson, San Jose, CA, USA) with 525 nm and 620 nm channels for SYTO 9 and PI fluorescence detection, respectively. Data acquisition was set to 50,000 events for each sample. Suspensions of untreated and 70% isopropyl alcohol-treated cells served as the negative control and the positive control, respectively.

### 4.6. Membrane Fluidity

Membrane fluidity was monitored by measuring fluorescence polarization of 1,6-diphenyl-1,3,5-hexatriene (DPH; Life Technologies) as previously reported with minor exceptions [31]. Briefly, logarithmic phase *S. aureus* ATCC 6538 cells were washed and resuspended in 0.85% sterile saline at a cell density of 1 × 10^9^ CFU/mL. The bacterial suspensions were incubated with different concentrations of CHQA at 25 °C for 1 h. Then, 1 × 10^−6^ M DPH was added to the above mixture, followed by further incubation for 30 min in the dark. Fluorescence polarization measurements were carried out on a Multi-Mode microplate reader (Synergy H1, Biotek Co., Winooski, VT, USA) equipped with 360/40 nm fluorescence excitation filter and 460/40 nm fluorescence emission filter. Suspensions of unlabeled cells with the same concentration served as reference blanks to subtract the excitant light scattering and other nonspecific contributions to the fluorescence signal. The polarization value is calculated by the following formula:

*P* = (*I*_VV_ − *GI*_VH_)/(*I*_VV_ + *GI*_VH_) (1)

where *I*_VV_ and *I*_VH_ are the fluorescence intensities emitted in the vertical and horizontal directions when the excitation beam is oriented vertically, respectively, and *G* is the grating factor.

### 4.7. Membrane Protein

The membrane protein assay was performed according to our previous reported method [33]. Briefly, logarithmic phase *S. aureus* ATCC 6538 cell suspension in 0.85% sterile saline (1 × 10^9^ CFU/mL) was prepared as described above. The cell suspensions were treated with different concentrations of CHQA at 25 °C for 1 h. The emission spectra of the mixtures were scanned from 280 nm to 400 nm with an excitation wavelength at 258 nm on the fluorescence spectrophotometer referred to above.

### 4.8. Transmission Electron Microscope (TEM)

Logarithmic phase *S. aureus* ATCC 6538 cells grown in nutrient broth (1 × 10^9^ CFU/mL) were exposed to CHQA at a final concentration of 2 × MIC at 37 °C for 6 h. Following treatment, cells were collected, washed, and prepared for transmission electron microscopy as previously described [38]. The ultrathin sections were observed and photographed using a Tecnai G2F20S-TWIN transmission electron microscope (FEI Co., Hillsboro, TX, USA).

### 4.9. Statistical Analysis

All experiments were carried out in triplicate, and the data were expressed as mean ± SD. One-way analysis of variance and Duncan′s multiple range tests were performed to determine significant differences (*p* < 0.05) between the means on SPSS 19.0 software(IBM Co., Armonk, NY, USA)

## 5. Conclusions

This is the first report on the antibacterial activity and mechanism of action of CHQA. The results demonstrated that CHQA possessed moderate antibacterial activity against both Gram-positive and Gram-negative bacteria. It was verified that the antibacterial activity of CHQA against *S. aureus* was achieved by damaging the cytoplasmic membrane with a significant membrane hyperpolarization, a loss of membrane integrity, as well as severe morphological change. Furthermore, the membrane-disruptive action of CHQA could be partially attributed to the interaction of CHQA with membrane lipid and protein, which consequently caused an increase in membrane fluidity and conformational changes in membrane protein, eventually resulting in membrane dysfunction and cell death. In conclusion, the antibacterial mechanism of CHQA against *S. aureus* involved the disruption of the bacterial cytoplasmic membrane and interaction with membrane components. These findings indicated that CHQA might be considered as a promising candidate for the development of a natural food preservative. However, these in vitro studies are limited in their ability to assess the effective concentration of CHQA in actual foods because of complex food components. Therefore, the effects of different food ingredients such as starch, protein, and fat on the antibacterial efficacy of CHQA may require investigation. Moreover, further research on the toxicological and sensory effects of CHQA, as well as the other possible mechanisms of action involved in intracellular macromolecule synthesis is necessary for application in food.

## Figures and Tables

**Table 1:** The minimum inhibitory concentrations of 3-*p*-*trans*-coumaroyl-2-hydroxyquinic acid (**1**) and chlorogenic acid (**2**) against different microorganisms. MIC: minimum inhibitory concentration.

Microorganism	MIC (mg/mL)	
	1	2
Gram-negative bacteria		
*Escherichia coli* ATCC 11229	10	10
*Salmonella enterica* ATCC 6539	5	5
*Vibrio parahaemolyticus* ATCC 17802	5	5
Gram-positive bacteria		
*Bacillus cereus* ATCC 14579	2.5	2.5
*Clostridium perfringens* ATCC 13124	5	5
*Staphylococcus aureus* ATCC 6538	5	5
*Staphylococcus aureus* ATCC 25923	5	5
*Staphylococcus aureus* ATCC 29213	5	5
*Staphylococcus aureus* ATCC 27217	5	5
*Staphylococcus aureus* ATCC 29247	5	5
*Staphylococcus aureus* ATCC 9144	5	5
